# Comparative Analysis of Osteoblastic Responses to Titanium and Alumina-Toughened Zirconia Implants: An In Vitro Study

**DOI:** 10.3390/biom14060719

**Published:** 2024-06-18

**Authors:** Elham Saberian, Andrej Jenča, Rahman Seyfaddini, Andrej Jenča, Hadi Zare-Zardini, Adriána Petrášová, Janka Jenčová

**Affiliations:** 1 Faculty of Medicine, Klinika of Stomatology and Maxillofacial Surgery Akadémia Košice Bacikova, Pavol Jozef Šafárik University, 040 01 Kosice, Slovakia; 2 Klinika of Stomatology and Maxillofacial Surgery Akadémia Košice Bacikova, UPJS LF, 040 01 Kosice, Slovakiaandrej.jenca1@upjs.sk (A.J.);; 3 Faculty of Medicine, Pavol Jozef Šafárik University, 040 11 Kosice, Slovakia; 4 Department of Biomedical Engineering, Meybod University, Meybod 89616-99557, Iran

**Keywords:** osteoblastic response, oral implantation, titanium implant, zirconium implant, bone regeneration, cell viability, apoptosis

## Abstract

Introduction: Osteoblastic responses play a crucial role in the success of oral implants. Enhanced proliferation of osteoblast cells is associated with reduced cell mortality and an increase in bone regeneration. This study aims to evaluate the osteoblastic responses following oral implantation. Materials and Methods: Osteoblast stem cells were harvested and subsequently cultivated using cell culture techniques. The osteoblastic phenotype of the extracted cells was confirmed by examining the extracellular matrix. Cell morphogenesis on functionalized biomaterial surfaces was assessed through indirect immunofluorescence staining. The cellular response was investigated in the presence of two types of implant materials: titanium (Ti) and alumina-toughened zirconia (ATZ). Cell viability and apoptosis were quantitatively assessed using MTT assays and flow cytometry, respectively. Results: The survival of osteoblastic lineage cells was moderately reduced post-implantation. Viability in the Ti implant group remained at approximately 86%, while in the ATZ group, it was observed at 75%, which is considered acceptable. Moreover, there was a significant disparity in cell survival between the two implant groups (*p* < 0.05). Analysis of apoptosis levels at various concentrations revealed that the rate of apoptosis was 3.6% in the control group and 18.5% in the ATZ group, indicating that apoptosis or programmed cell death in the ATZ-treated group had increased nearly four-fold (*p* < 0.05). Conclusions: The findings of this study indicate a reduction in osteoblastic cell line survival following implant treatment, with titanium implants exhibiting superior performance in terms of cell survival. However, it was also noted that the incidence of apoptosis in osteoblast cells was significantly higher in the presence of zirconium-based implants.

## 1. Introduction

Dental implants have revolutionized the field of restorative dentistry as a gold standard for tooth replacement, offering a durable and functionally superior alternative to traditional prosthetic options [1]. The success of dental implants is largely contingent upon the phenomenon of osseointegration, a process characterized by the direct structural and functional connection between the living bone and the implant surface [2]. At the cellular level, this intricate process is orchestrated by osteoblasts, the bone-forming cells responsible for the synthesis and mineralization of the bone extracellular matrix [3]. The interplay between osteoblasts and implant materials, such as titanium and alumina-toughened zirconia (ATZ), is of paramount importance due to their distinct physicochemical properties, which can significantly influence cellular responses and, consequently, the quality of osseointegration [4,5].

Titanium has been the material of choice in dental implantology for several decades, owing to its exceptional biocompatibility, favorable mechanical properties, and proven track record of clinical success [6]. The biocompatibility of titanium is largely attributed to the formation of a passive, stable, and biologically active titanium oxide layer on its surface, which promotes the adhesion, proliferation, and differentiation of osteoblasts, thereby facilitating the formation of a robust bone–implant interface [7,8]. Moreover, titanium implants have been shown to elicit a minimal inflammatory response and are associated with a lower incidence of peri-implant diseases, such as mucositis and peri-implantitis, which can compromise implant stability and longevity [9,10].

In recent years, ATZ has emerged as a promising alternative to titanium, particularly for patients with aesthetic concerns or metal sensitivities. ATZ offers several advantages over titanium, including superior aesthetic qualities, lower plaque affinity, and similar mechanical properties. However, the biological responses of osteoblasts to ATZ implants are not yet fully understood, necessitating a comprehensive investigation to elucidate their potential benefits and limitations in clinical practice [11,12].

The surface characteristics of dental implants, including surface chemistry, roughness, and topography, play a pivotal role in modulating osteoblast behavior and osseointegration outcomes. Roughened implant surfaces have been shown to enhance osteoblast adhesion, proliferation, and differentiation, thereby promoting the formation of a thicker and stronger bone–implant interface. Furthermore, the initial inflammatory response and the risk of peri-implant diseases are influenced by the implant material, with certain materials eliciting a more favorable tissue response and reducing the incidence of complications [13,14].

In light of the critical role of osteoblasts in bone formation and implant success, the primary objective of this study is to conduct a comparative analysis of osteoblastic responses to titanium and ATZ implants. By investigating the early-stage proliferation, differentiation, and cytotoxicity of osteoblasts in contact with these materials, we aim to provide valuable insights into their respective capabilities to support osseointegration and inform future material selection in implant dentistry [15,16]. The findings of this study have the potential to contribute to the optimization of implant surface designs and the development of innovative materials that can enhance clinical outcomes and improve patient satisfaction.

In conclusion, the selection of implant materials has a profound impact on the biological processes underlying osseointegration and the long-term success of dental implants. By gaining a deeper understanding of the osteoblastic responses to titanium and ATZ implants, this study aims to comparatively analyze osteoblastic responses to titanium and alumina-toughened zirconia implants.

## 2. Materials and Methods

### 2.1. Preparation of Osteoblast Cells

This in vitro study, conducted from November 2023 to February 2024, aimed to evaluate the osteoblastic response following oral implantation. Sample collection involved a 50 mL Falcon tube containing 4 mL serum-free DMEM-F12 medium, 120 μL penicillin-streptomycin, and fungizone, transported to the cell culture laboratory at temperatures below 10 °C [17].

### 2.2. Implant Materials and Surface Treatments

For cell culture experiments, 20 mm diameter, 1.5 mm thick biomaterial discs were fabricated from alumina-enhanced zirconia (ATZ) (BIO-HIP^®^, Metoxit, Thayngen, Switzerland) and grade 4 titanium (Ti) (Nobel Biocare^®^, Kloten, Switzerland), both commonly utilized implants. To stimulate osseointegration, selected surfaces were treated: (i) the micro-porous ZircaPore^®^ coating on ATZ and (ii) TiUnite^®^ surface via electrochemical anodization creating a porous topography on titanium discs. The implant surfaces were not further processed to retain machining characteristics representative of transgingival implant parts, including ATZ and Ti as controls without biomaterial layers [18].

### 2.3. Cell Surface Characterization

A scanning electron microscope (Jeol^®^, Freising, Germany) was used to examine and determine the morphology and diameter of the designed implant structure together with the osteoblast cells. For this purpose, the samples were covered with a very thin layer of fibroblast cells to assess the effect of the implants. The images were taken at different magnifications. To study the diameter distribution of the nanofibers, the diameter of a series of randomly selected structures was measured using ImageJ software (version 1.53k) and the results were expressed as an average. To investigate the morphology of the cells, the cells were first fixed on the surface with glutaraldehyde. In the next step, all samples were dehydrated with ethanol at different percentages and placed in the environment to dry the solvents completely. Then the samples were analyzed with the SEM [19].

### 2.4. Isolation of Mononuclear Cells from Tissue

To isolate osteoblast cells, the root tissue sample was first washed in five Petri dishes with 4 cc PBS (Invitrogen^®^, Karlsruhe, Germany), 120 microliters of Penstrep and Fungizone to remove lesions and blood. Then the samples were divided into small pieces and poured into a 15 mL Falcon with 1 cc Dispase, 3 cc HBSS and 80 microliters Penstrep and Fungizone. This was kept at a temperature of 4 degrees for 12–14 h to separate the osteoblasts from the root tissue. The isolated osteoblasts were completely crushed on one side with 2 cc of PBS using a scalpel and added with the same amount of 0.25% trypsin and placed in the incubator for 15 min. After this time, 10% DMEM was added and pipetted, then the cells were centrifuged. The discarded supernatant was added to 2 cc of DMEM-F12. The supernatant solution was discarded, and 2 cc of Epilife was added to the cells and the cells were counted. After microscopic observation and confirmation of the number of cells by counting and their correctness, the flask was placed in the incubator. To investigate the nature of the osteoblast stem cells, the percentage of expression of a range of mesenchymal stem cell surface antigens was assessed [20].

### 2.5. Cell Culture

Osteoblast cells derived from human oral tissue (HOB) were cultured in Dulbecco’s Modified Eagle’s Medium supplemented with 0.2% (*w*/*v*) kanamycin (Sigma-Aldrich^®^, Taufkirchen, Germany), 10% (*w*/*v*) fetal calf serum (Biochrom^®^ AG, Berlin, Germany) and 1% (*w*/*v*) glutamine. After culturing the desired cells, the cells were maintained in a humidified 36 °C incubator with 5% CO_2_. The osteoblastic phenotype of the isolated cells was verified by the mineralization potential of the extracellular matrix (ECM) of the confluent cell cultures at day 28, as described in previous studies [21,22]. Osteoblasts were passaged after reaching approximately 70% confluence. All experiments in this study were performed with osteoblasts of passages 5 and 6. For cell culture on the biomaterial slices, 1 × 10^4^ cells/mL per slice were seeded in a 12-well plate (cell density of 0.28 × 10^4^/cm^2^).

### 2.6. Morphogenesis

Cell morphogenesis on functionalized and non-functionalized biomaterial surfaces was investigated by indirect immunofluorescence of phalloidin labelling of actin at day 3 of cell culture. In brief, for fluorescence microscopy, cells were fixed with 4% formaldehyde in phosphate-buffered saline (PBS) for 20 min at room temperature. Actin labelling was performed by incubating the samples for 30 min with phalloidin conjugate (TexasRed™-X Phalloidin^®^, ThermoFisher, Waltham, MA, USA) diluted 1:40 in PBS containing 0.5% BSA (*w*/*v*). Nuclei were stained with 0.001% Hoechst 33,342 (Invitrogen^®^, Dreieich, Germany) in 0.5% BSA in PBS for 15 min. Optical analysis was performed using the RB50 fluorescence microscope (HS Code^®^, Zhangjiagang, China). Quantitative morphometric analysis of the fluorescence microscope images was performed using the microscope software (version 2.2, KEYENCE) after 3 days of cell culture on the biomaterials. The morphology of as many cells as possible (140 ≤ n ≤ 370, 2 independent experiments with 2 biomaterial slices per subgroup) was analyzed as described previously by measuring the cell area, cell perimeter, major axis representing the long axis of the smallest rectangle drawn around the cell body, and the minor axis indicating the width of the rectangle [23,24].

### 2.7. Investigation of the Implant on the Proliferation of Osteoblast Cells

To investigate the effect of the purified dual-specific antibody on cell proliferation, the MTT assay was performed according to the standard method. This experiment was performed with the HOB cell line. On the first day of the experiment, two T-flasks, each of which had already been cultured with the strains mentioned, were exposed to the effect of the enzyme trypsin. The cells of each strain were poured into a 15 mL Falcon, and 5 mL culture medium was added to each. Complete cells (MDEM) were added. Before centrifugation, the number of cells in each flask was counted using a blood cell counting slide (hemocytometer) in a sterile environment. The number of cells per millimeter was then reported. After counting the cells, they were centrifuged at 1200 rpm for 5 min. Then the supernatant was discarded and the cell pellet was redissolved by adding the same volume of culture medium that was added before centrifugation, i.e., 5 mL. Then, 7000 or 10,000 HOB cells for each well were cast into 96-well plates. Based on this number of cells for each well, the total number of cells required for the wells was calculated according to the type, and the required volume was selected from the cultured cells. Then, 200 microliters of cells were added to each well and incubated overnight. They were incubated in a culture incubator. On the second day, after the cell culture medium (Life Technologies^®^, Darmstadt, Germany) was emptied into each well (it should be noted that a special plate centrifuge was used for the deposition of non-adherent cells such as Jurkat, and after sedimentation of the cells, the supernatant solution was discarded), 200 microliters of medium A of fresh cell culture was added to each well. At this point, the volume of solution in each microtube was 600 µL, which was sufficient for three replicates. On the third day, 50 microliters of a 2 mg/mL MTT solution was added to each well and incubated at 37 °C for 4 h. Meanwhile, no antibody was added to a group of three wells, i.e., the twelfth row. Then, 200 μL DMSO was added to each well to dissolve the formazan. The plate was then covered and shaken on a shaker for 15 min. The plate was then covered and the wells were read at a wavelength of 570 nm and a reference wavelength of 650 nm [25,26].

### 2.8. Cell Death Assay by Implant

The study of cell death induced by the target antibody was performed using the HOB cell line, with two discs for each experimental run. On day one, one million cells from each line were cultivated in two T25 flasks. After a 48-h incubation period, the supernatant was collected, and the cells were detached from their substrate using trypsin^®^. The isolated cells were then washed twice with sterile phosphate-buffered saline (PBS^®^) through centrifugation at 1200 rpm for 5 min. Prior to centrifugation, a hemocytometer^®^ was employed for cell counting. The cell pellet was resuspended in 2 mL of PBS buffer. For each cell line, three tubes were prepared: a test tube, an unstained control tube, and an annexin and propidium iodide-stained tube, each receiving 400 μL of cells. The test tube was treated with 2 μL of annexi^®^ (1 mg/mL), 2 μL of propidium iodide^®^ (1 mg/mL), and 100 μL of incubation buffer. The first control tube contained only 100 μL of incubation buffer^®^ and 400 μL of cells, the second control tube had 2 μL of annexin^®^ and 100 μL of incubation buffer, and the third control tube comprised 100 μL of propidium iodide^®^ in 400 μL of cells. Cell death was assessed using a flow cytometer. The impact of purified recombinant antibody on inhibiting cell migration and invasion was examined following the protocol of the BME Medium Well Culture Coat 96 kit^®^ [27,28].

### 2.9. Data Analysis

Statistical analysis was performed using SPSS software (version 26). One-way analysis of variance (ANOVA) was employed to compare the effects of the implant between experimental groups. All data are presented as mean ± standard deviation. Statistical significance was determined at a *p*-value of less than 0.05.

## 3. Results

### 3.1. Microscopic Evaluation of Osteoblast Cells

**Figure 1:**
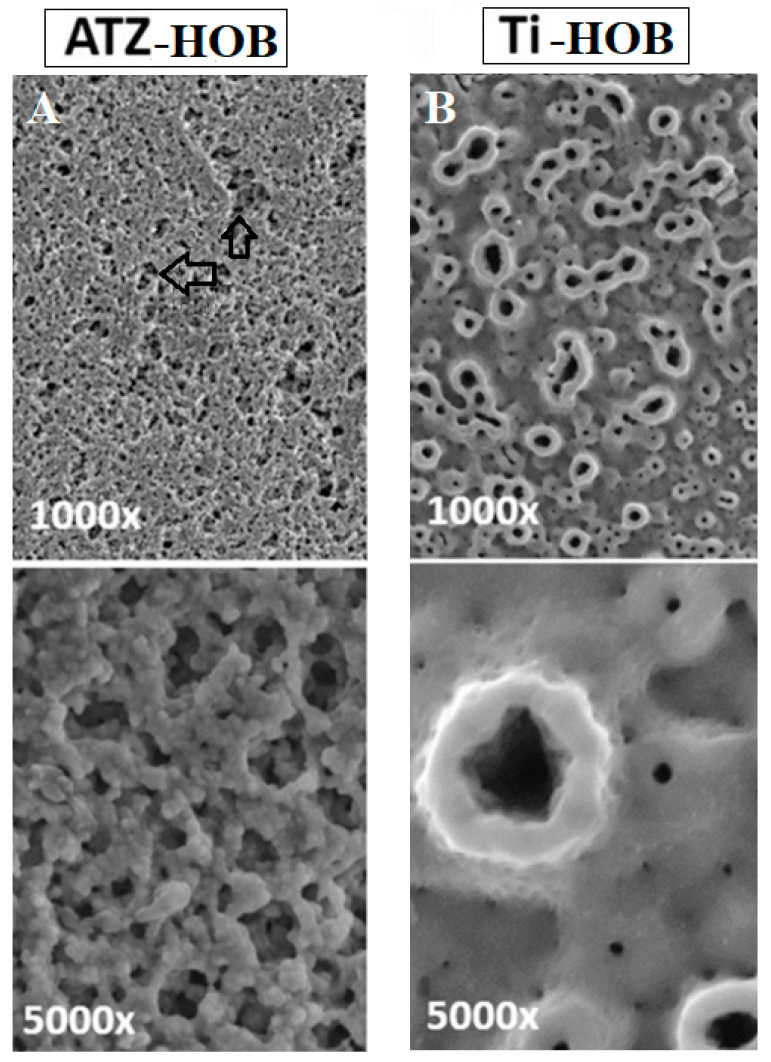
Electron microscope image with different magnification. ATZ (**A**), and titanium (**B**) They are visible on the surface of osteoblast cells. Magnifications were set to 1000× (first row) and 5000× (second row). Arrows point to macroscopic grooves and gaps on ceramic surfaces.

The microscopic examination of osteoblast cells was conducted using scanning electron microscopy (SEM) to assess the impact of surface roughness on the hybrid osteoblast (HOB) culture. The SEM images revealed profound disparities between the microporous alumina-toughened zirconia (ATZ)-based ZircaPore^®^ surfaces and the electrochemically anodized titanium discs (Figure 1), with statistically significant differences observed in surface topography (*p* < 0.05).

In the case of the ATZ-based ZircaPore^®^ surfaces, a distinct and complex microstructure was observed. These surfaces featured interconnected micropores, which were accompanied by intermittent macroscopic grooves and gaps, as indicated by the arrows in Figure 1a. Upon closer inspection at a higher magnification (Figure 1B, bottom row), the zirconia coating displayed a granular texture, suggesting a potentially more favorable environment for cell adhesion and proliferation.

In contrast, the titanium discs exhibited a different surface morphology. As depicted in Figure 1B, the titanium surfaces were characterized by smaller pores with diameters ranging approximately from 4 to 12 μm. These pores were irregularly scattered across the surface, creating a less uniform topology. The edges of the pores and the interstitial spaces between them appeared smooth, in the order of a few micrometers, which may influence cellular behavior and attachment.

SEM analysis further confirmed that there were no discernible differences in surface topography between the plasma-functionalized titanium surfaces and the control group. This indicates that the plasma treatment did not significantly alter the surface characteristics at the microscopic level, preserving the initial pore structure and distribution.

These findings highlight the unique topographical features of both ATZ ZircaPore^®^ and titanium surfaces, which could potentially affect osteoblast behavior, proliferation, and the overall osseointegration process. The differences in surface roughness and porosity might play a crucial role in determining the cellular response and the success of dental implants.

### 3.2. Cell Morphology of Osteoblasts

**Figure 2:**
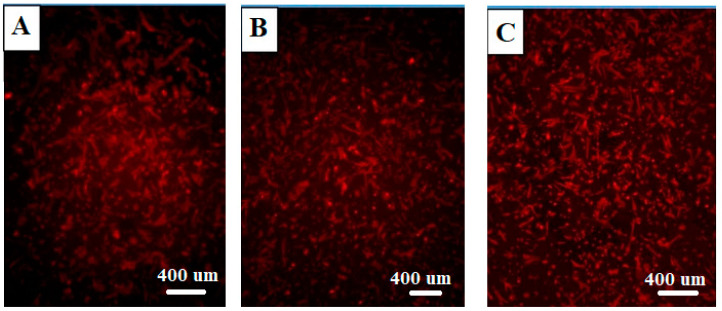
Osteoblast cells were examined on the third day of culture after implant placement with the help of immunofluorescence of phalloidin-labeled actin (red fluorescence). (**A**) ATZ zirconium implant group, (**B**) Ti group under titanium implant, (C) control.

The cell morphology of osteoblasts was analyzed using fluorescence microscopy, specifically by staining actin and vinculin to assess cell density and cytoskeleton organization. At the third day of cell culture, the fluorescent images were captured to reveal the actin cytoskeleton (labeled with red fluorescence) and provide insight into the cell distribution across the biomaterial surfaces (Figure 2).

As depicted in Figure 2, there was a similar cell density observed on both the functionalized surfaces and the control samples at all-time points assessed. This demonstrates that the surface modifications did not significantly affect the initial attachment and spreading of the cells on the tested materials.

The results from this analysis exposed a material- and time-dependent organization of the cytoskeleton. The osteoblasts on ceramic (ATZ ZircaPore^®^) surfaces exhibited a less pronounced formation of actin tension fibers and delayed vinculin expression compared to those on titanium surfaces. Vinculin, a focal adhesion protein, is critical for cell–matrix interactions and plays a role in cell migration and cytoskeletal organization. The delayed expression of vinculin on ceramic surfaces suggests that the cells might be adapting to the unique characteristics of these surfaces, potentially requiring more time to establish strong attachments.

Despite these differences in cytoskeletal organization, the overall morphology of the osteoblast cells was found to be quite similar across the investigated groups. This implies that while the cells might be adapting to the distinct surface properties, they maintain a consistent shape and structure, which is essential for their primary functions in bone formation and maintenance.

### 3.3. Effects of the Implants on Survival

**Figure 3:**
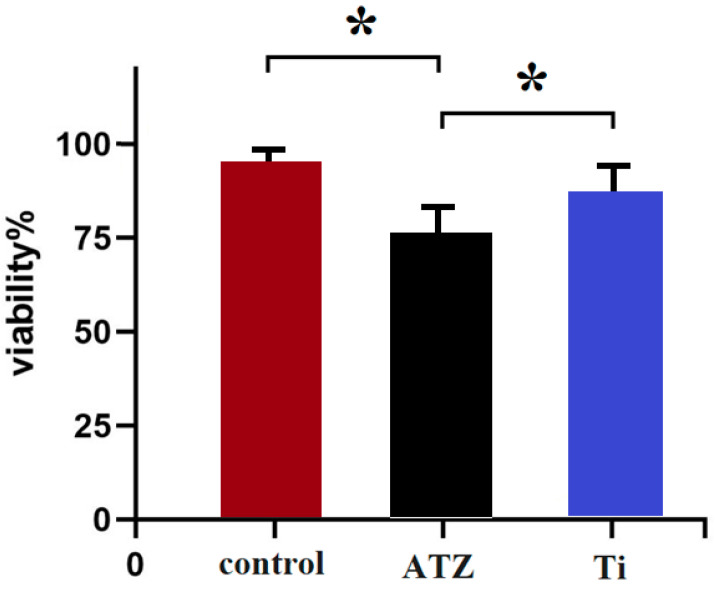
The results related to the effect of the implant on the induction of cell death in osteoblast cells. ATZ group of zirconium implants, Ti group under titanium implants. Asterisks indicate statistically significant differences between groups (*p* < 0.05).

The impact of the dental implants on the survival rate of osteoblast cells was investigated to evaluate any potential cytotoxic effects. The findings revealed that following exposure to the implants, there was a slight decrease in cell survival. When the osteoblast cells were treated with the titanium (Ti) implant, the survival rate was approximately 86%, indicating that a majority of the cells remained viable after the treatment (Figure 3).

For the ATZ implant, the cell survival rate was slightly lower at around 75%. Despite this decrease, both survival rates, 86% for Ti and 75% for ATZ, were considered to be within an acceptable range for cell viability (Figure 3). However, a statistically significant difference (*p* < 0.05) was noted between the two implant materials, with titanium demonstrating better cell survival outcomes.

These results imply that neither implant induced cytotoxic effects on the osteoblast cells, as the survival rates were not significantly detrimental. Nevertheless, titanium implants appeared to promote a more favorable environment for osteoblast survival compared to the ATZ implants (*p* < 0.05). This suggests that titanium might be more biocompatible in terms of maintaining cell viability, which is a critical factor in the osseointegration process and overall success of dental implants.

### 3.4. Investigation of Cell Proliferation and Cell Death

**Figure 4:**
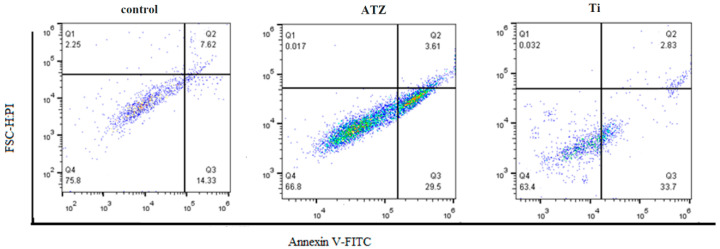
Evaluation of cell death and proliferation with the help of flow cytometry in the examined groups (ATZ group under zirconium implant, Ti group under titanium implant): according to the Q4 area, the live cells fluctuated between 63 and 75%.

The investigation of the effect of the implant on the osteoblast cell lines after three days showed the suitability for the proliferation of osteoclast cells. According to the flow cytometry data, 66.8 osteoblast cells were alive in the zirconium group and 63.4 in the titanium group (Figure 4).

**Figure 5:**
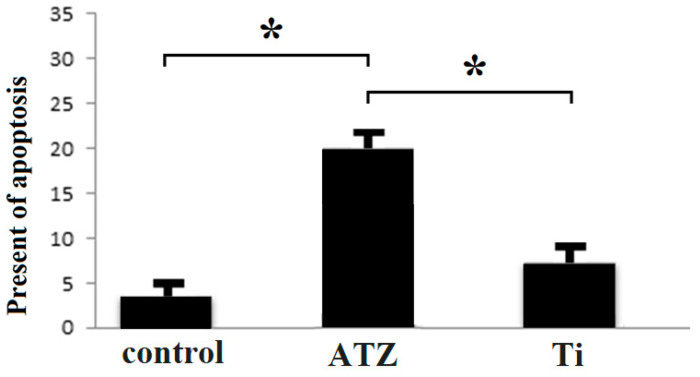
Investigation of the amount of apoptosis in the two investigated groups. Asterisks indicate statistically significant differences between groups (*p* < 0.05).

Examination of apoptosis levels at various concentrations using annexin apoptosis assays revealed that the percentage of apoptotic cells was 3.6% in the control group and 18.5% in the ATZ-treated group. This indicates that the rate of apoptosis, or programmed cell death, increased nearly four-fold in the group treated with ATZ. These findings demonstrate that, in an experimental setting, zirconium-based implant treatment significantly elevates the level of programmed cell death among osteogenic cells (Figure 5).

## 4. Discussion

Considering the importance of osteoblast cells in the implant, the present study investigated the response of osteoblast cells of two types of implants. The study conducted utilizes SEM to explore the impact of surface roughness HOB culture over different substrates, specifically ATZ-based ZircaPore^®^ surfaces and electrochemically anodized titanium discs. This analysis is pivotal in understanding how surface topography influences cell behavior, particularly in the context of dental implants, where osseointegration and cell adhesion are critical for success [29].

The SEM images of ATZ-based ZircaPore^®^ surfaces revealed a complex microstructure characterized by interconnected micropores and macroscopic grooves/gaps. This intricate pattern is hypothesized to create a more favorable environment for cell adhesion and proliferation. The granular texture of the zirconia coating at higher magnifications suggests a surface that could potentially enhance cellular interactions, a finding supported by previous research, indicating that rough surfaces with specific topographical features promote cell adhesion and proliferation [30].

In contrast, the titanium discs presented with smaller, irregularly scattered pores across their surface, creating a less uniform topology. The smoothness of the pores and interstitial spaces, as observed, could influence cellular behavior and attachment differently compared to the ATZ-based ZircaPore^®^ surfaces. This variation in surface morphology may affect the osseointegration process, with implications for the success of titanium-based dental implants [31].

The SEM analysis further elucidated that plasma treatment did not significantly alter the surface topography of titanium surfaces, preserving the initial pore structure and distribution. This finding is consistent with studies that have shown plasma treatment to primarily affect surface chemistry rather than topography [32].

The distinct surface roughness and porosity observed between ATZ ZircaPore^®^ and titanium surfaces suggest that these physical attributes play a crucial role in determining osteoblast behavior and the overall osseointegration process. Surface roughness, through its influence on cell adhesion and proliferation, is a critical factor in the success of dental implants. Research has consistently shown that surface modifications aimed at increasing surface roughness and porosity can enhance the osseointegration process [33,34].

The analysis of osteoblast cell morphology using fluorescence microscopy, with specific staining for actin and vinculin, provides valuable insights into cell density, cytoskeleton organization, and the adaptation of cells to different biomaterial surfaces. The findings, as depicted in Figure 2, suggest a nuanced interaction between osteoblasts and the surface properties of ATZ ZircaPore^®^ ceramic and titanium materials.

The observation of similar cell density on both functionalized surfaces and control samples at all time points indicates that the surface modifications did not significantly impact the initial attachment and spreading of osteoblasts. This suggests the fundamental compatibility of these materials with osteoblast behavior, which is crucial for applications in bone tissue engineering and dental implants [35].

The material- and time-dependent organization of the cytoskeleton, particularly the less pronounced formation of actin tension fibers and delayed vinculin expression on ceramic surfaces compared to titanium, hints at a more complex adaptation process. Vinculin, as a key focal adhesion protein involved in cell–matrix interactions, cell migration, and cytoskeletal organization [30], plays a critical role in the cellular response to surface topography. The delayed expression of vinculin on ceramic surfaces may reflect the cells’ need for additional time to establish robust attachments to the unique characteristics of these surfaces, potentially influencing the dynamics of cell migration and differentiation [36].

Despite these differences in cytoskeletal organization, the maintenance of a consistent overall morphology across the investigated groups underscores the adaptability of osteoblasts to distinct surface properties while preserving their essential functions in bone biology. This adaptability is crucial for the success of biomaterials in orthopedic and dental applications, where the integration of materials with bone tissue is paramount [37].

The investigation into the impact of dental implants on the survival rate of osteoblast cells provides crucial insights into the potential cytotoxic effects and biocompatibility of the materials used. The findings, which indicate a slight decrease in cell survival following exposure to the implants, underscore the importance of assessing material safety and compatibility with cellular environments.

The observed difference in osteoblast survival rates following treatment with the titanium (Ti) and ATZ implants underscores the subtle yet important variations in biocompatibility between these materials. Although the majority of cells remained viable after treatment with either implant type, the titanium implant consistently demonstrated a higher survival rate compared to the ATZ implant. This suggests that while neither material exhibited overt cytotoxic effects, the titanium implant provided a more conducive environment for osteoblast survival. This finding carries significant implications for the osseointegration process, as enhanced osteoblast viability is crucial for successful implant integration and long-term stability [35].

The implications of these findings are significant for the field of dental implantology. Titanium, already a widely used material in dental and orthopedic implants due to its excellent biocompatibility and mechanical properties, is further validated by these results. The higher cell survival rate suggests that titanium may offer superior conditions for cell viability and proliferation, which are essential for the integration of implants with the surrounding bone tissue [36].

However, it is important to note that the survival rates for both materials were considered within an acceptable range for cell viability. This indicates that while titanium may offer slight advantages in terms of cell survival, ATZ implants also present a viable option with satisfactory biocompatibility. The choice between these materials may depend on various factors, including specific clinical requirements, mechanical properties, and potential advantages in terms of infection resistance or aesthetic outcomes.

The investigation into the effect of the implant materials on osteoblast cell lines after three days provides important insights into the suitability of these materials for supporting cell proliferation and the potential induction of cell death. The flow cytometry data, indicating 66.8% live osteoblast cells in the zirconium (ATZ) group and 63.4% in the titanium group, suggests that both materials are conducive to cell survival and proliferation. However, the slight difference in live cell percentages may indicate a marginally better performance of zirconium in supporting cell viability, at least within the context of this study.

The examination of apoptosis levels using annexin apoptosis assays reveals a significant difference in the rate of programmed cell death between the control group (3.6% apoptotic cells) and the ATZ-treated group (18.5% apoptotic cells). This nearly four-fold increase in apoptosis in the ATZ-treated group suggests that zirconium-based implant treatment may have a more pronounced effect on inducing programmed cell death among osteogenic cells. This finding is crucial as it points to a potential limitation of zirconium-based implants in maintaining long-term cell viability and function, which is essential for successful osseointegration and implant longevity.

These results underscore the complex interplay between biomaterials and cellular responses. While zirconium may offer certain advantages in terms of initial cell survival and proliferation, the elevated levels of apoptosis observed with this material raise concerns about its long-term biocompatibility and the potential for inducing cell death pathways that could compromise implant success.

In contrast, titanium, despite showing a slightly lower percentage of live cells in the flow cytometry analysis, appears to have a lower impact on inducing apoptosis, suggesting a more favorable profile in terms of maintaining cell viability over time. This is consistent with the well-documented biocompatibility of titanium and its widespread use in dental and orthopedic implants [37].

The results showed that after the cells were treated with the implant, the survival of the osteoblast lineage cells decreased to a certain extent. In the sample of Ti implant, the percentage of time was 86% and in ATZ was 75%, which was acceptable. In addition, a significant difference was found between the implant samples studied in terms of survival. The results suggest that the implants have no toxicity in terms of osteoblast cell survival and the titanium implant performed better in terms of survival.

It is reported that titanium is a very reactive metal that oxidizes in a very short time when exposed to air and that it has a high resistance to kerogen due to its inactive oxidized layer. These properties of the implant surface have been fully demonstrated in an effort to achieve better bone anchorage [38]. In a study on the putative effect of plasma-functionalized implant biomaterials on oral tissue cells, the response of osteoblast cells to zirconia- and titanium-based implant surfaces was investigated. The results showed that plasma functionalization increased the wettability of the implant surface [39]. According to studies, implants naturally cause cytotoxicity by releasing titanium compounds to the surrounding cells. Particle size influences the toxicity of titanium [40].

The biocompatibility of the implant is very important for the gums. The toxicity of the materials used for the implant may play a role in cell death and inflammation [41]. In the present study, the percentage of proliferation was at an appropriate level in the groups studied. Toxicity was minimal.

In addition, the study of the apoptosis percentage at different concentrations showed that the death of bone-forming cells was significantly increased in the zirconium group after treatment with the implant. Titanium implants with a low modulus of elasticity have excellent biocompatibility and cause rapid bone formation at the bone-implant interface. In fact, the increase in the surface area of the bone–implant interface with titanium implants allows for rapid bone growth and improved bone integrity [42].

Various studies show that several factors influence the dental implant and its osseointegration. In the studies conducted on the nature and type of metal implants, some are coated or polished [43,44]. Osteoblast cells play an important role in osteogenesis. When osteoblast cells increase, cell death decreases and bone growth increases. Osseointegration is the process by which a dental implant fuses with the jawbone. The implant and the bone fuse together, creating a strong and stable foundation for the replacement tooth. This process, which can take several months, is crucial for the stability and functionality of the dental implant [31]. Osseointegration is crucial for the success of dental implants. By integrating the implant into the surrounding bone tissue, osseointegration provides stability, prevents implant failure, promotes oral health and improves aesthetics [45].

In the current study, titanium showed better results from a biological point of view. In general, titanium is a suitable metal for dental treatments. Microscopic evaluation of the osteoblast cells showed differences between the surface of two types of zirconium and titanium implants. The morphology of the osteoblast cells was very similar in the groups studied.

One of the ultimate therapeutic goals is the complete restoration of the root of the damaged periodontal attachment system, and because the retrophilic materials are in direct contact with living tissues such as connective tissue and bone. The actual reconstruction of this tissue depends on the correct function of osteoblasts, fibroblasts and cementoblasts to restore bone, PDL and cementum. Therefore, it is necessary to use materials that are ideal in terms of tissue compatibility and cell toxicity and have the ability to stimulate healing in this area [46]. Of course, in addition to the cytotoxicity and non-allergenicity of the implant composition, the use of materials that promote bone formation can also play an effective role in improving the quality of treatment [47].

It should be noted that osteoblast and osteoclast cells exist in a normal state. However, in the case of tissue inflammation, osteoclasts proliferate more and osteoblasts decrease, which has a negative effect on ossification and osteogenesis [48].

One of the limitations of the present study was to investigate the effects of some clinical factors. One factor that influences the implant is the age of the patients. In the laboratory, factors such as the patient’s age and condition cannot be tested, whereas in vivo, age and condition are effective factors for the implant [49]. One of the strengths of current research is the study of the molecular and biological factors that are effective for the growth and proliferation of osteoblast cells.

In summary, the analysis of osteoblast cell morphology using fluorescence microscopy revealed similar cell densities on both functionalized surfaces and the control, suggesting that surface modifications did not significantly impact cell attachment and spreading. However, a material- and time-dependent cytoskeleton organization was observed, with osteoblasts on ceramic surfaces showing reduced actin tension fiber formation and delayed vinculin expression. This indicates that osteoblasts might adapt to the unique properties of ceramic surfaces, possibly requiring more time to establish robust attachments. The study also found that both implant materials showed acceptable survival rates for osteoblast cells, with titanium implants demonstrating significantly better survival outcomes (86%) compared to ATZ implants (75%). This suggests that titanium might be more biocompatible for maintaining cell viability, which is crucial for successful osseointegration. Flow cytometry analysis further confirmed the proliferation potential of osteoblasts, with slightly more live cells in the titanium group compared to the zirconium group. However, the ATZ group exhibited a four-fold increase in apoptosis or programmed cell death, indicating that osteoblasts may be more susceptible to cell death with ATZ implants. These results collectively imply that while both implant materials support osteoblast cell survival and proliferation to some extent, titanium appears to be more advantageous, particularly in terms of cytoskeleton organization, cell survival, and reduced apoptosis. The differences in surface properties and cellular responses might be crucial in determining the long-term success and biocompatibility of dental implants. Further in vitro and in vivo studies are warranted to validate these findings and explore potential surface modifications to optimize the performance of ATZ implants.

## 5. Conclusions

In conclusion, this study highlights the unique topographical features of both ATZ-based ZircaPore^®^ and titanium surfaces, which may affect the behavior of osteoblast cells and the osseointegration process. The differences in surface roughness and porosity might play a crucial role in determining the cellular response. While both materials showed acceptable cell viability, titanium appeared to promote a more favorable environment for osteoblast survival. These findings suggest that the choice of implant material may influence the success of dental implants, and further research is needed to fully understand the interactions between implant surfaces and osteoblast cells.

## Data Availability

Data can be made available on request.
